# Coronary artery distensibility assessed by cardiovascular magnetic resonance imaging in patients with type 2 diabetes mellitus and healthy controls

**DOI:** 10.1186/1532-429X-15-S1-M5

**Published:** 2013-01-30

**Authors:** David Jean Winkel, Tingting Xiong, Nikolaus Tiling, Matthias Stuber, Allison Hays, Robert G Weiss, Rolf Gebker, Eckart Fleck, Ursula Plöckinger, Sebastian Kelle

**Affiliations:** 1 Internal Medicine/Cardiology, German Heart Institute Berlin, Berlin, Germany; 2 Interdisziplinäres Stoffwechsel-Centrum, Charité-Universitätsmedizin Berlin, Campus Virchow-Klinikum, Berlin, Germany; 3 Department of Medicine, Division of Cardiology, Johns Hopkins University, Baltimore, MD, USA; 4 Department of Radiology, Division of Magnetic Resonance Research, Johns Hopkins University, Baltimore, MD, USA; 5 Department of Biomedical Engineering, Johns Hopkins University, Baltimore, MD, USA; 6 Department of Radiology, Centre Hospitalier Universitaire Vaudois, Center for Biomedical Imaging (CIBM), University of Lausanne, Lausanne, Switzerland

## Background

Recently, measurement of coronary artery distensibility by MRI has been demonstrated [1,2]. We sought to assess coronary artery distensibility non-invasively in older healthy subjects and patients with type 2 diabetes mellitus (DM), and to analyze differences in coronary artery distensibility in patients with DM based on the presence or absence of coronary artery disease (CAD).

## Methods

A total of 29 patients with DM treated with insulin (20 men, mean age 62 ± 10 years, mean ±SD) and 10 healthy, adult subjects (4 men, mean age 54 ± 4 years) were studied using a commercial whole-body 3.0 Tesla MRI system. In 13 (45%) patients with diabetes CAD was known (mean age 62 ± 10 years); in 16 (55%) DM patients CAD was absent (mean age 62 ± 11 years). The presence of CAD was defined using a previous coronary x-ray angiogram. In each subject, the proximal segment of a coronary artery was imaged for cross-sectional area measurements using cine spiral MRI [3]. Distensibility (mmHg-1*103) was determined as (lumen max - lumen min)/(pulse pressure x lumen min) x 1000. The pulse pressure was calculated as the difference between the systolic and diastolic brachial blood pressure. All continuous parameters are given as mean + one standard deviation (SD). For all tests, p<0.05 was considered statistically significant. All tests were two-sided.

## Results

**Figure 1:**
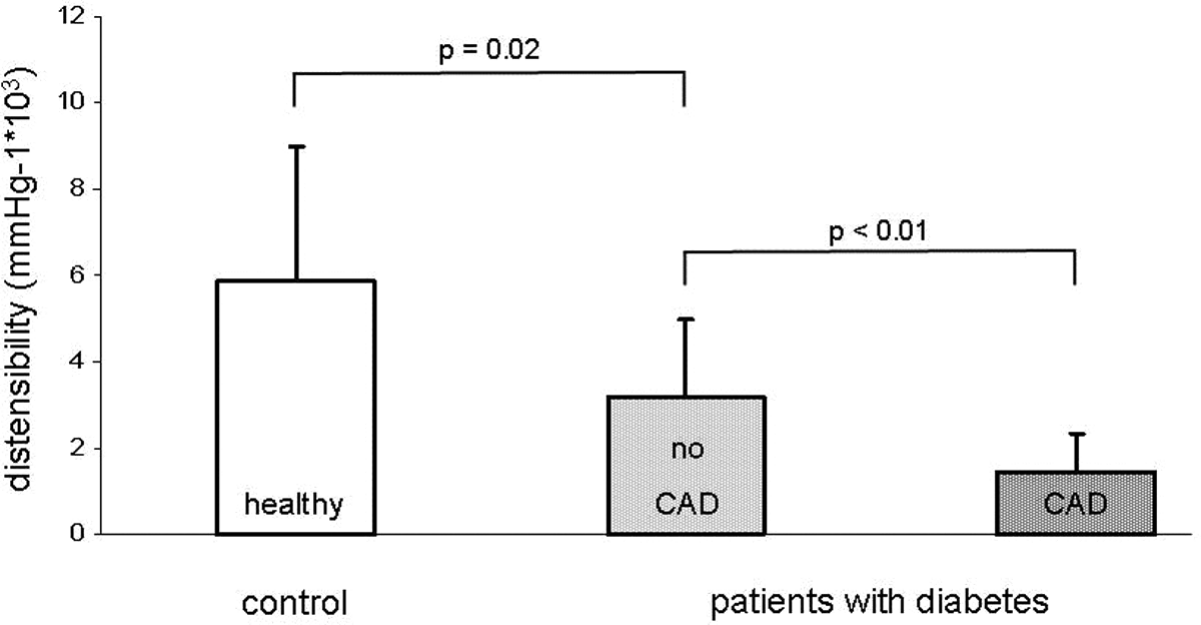


A total of 23 patients (24 coronary artery segments) with type 2 diabetes mellitus and 10 healthy subjects (13 coronary artery segments) had adequate image quality for coronary area measurements. Coronary artery distensibility was significantly higher in the healthy subjects than in those with DM only (5.9 ± 3.0 vs. 3.2 ± 1.8 mm Hg-1*103, p = 0.02; median 5.5 vs. 3.5) and higher in patients with DM only than in patients with both DM and CAD 3.2 ± 1.8 vs..1.4 ± 0.9, p < 0.01, median 3.5 vs. 1.4), see Figure 1.

## Conclusions

Coronary artery distensibility is significantly higher in healthy controls than in patients with DM. Our non-invasive measurements suggest that the presence of low coronary artery distensibility in patients with DM is associated with CAD.

## Funding

none

## References

[B1] Kelle Am J Cardiol 2011 108 4 491 7 10.1016/j.amjcard.2011.03.078 21624552 PMC3159191

[B2] Lin Radiology 2011 261 3 771 8 10.1148/radiol.11110573 21875853 PMC3219916

[B3] Hays J Am Coll Cardiol 2010 56 20 1657 65 10.1016/j.jacc.2010.06.036 21050976

